# Neuroimmune semaphorins as costimulatory molecules and beyond

**DOI:** 10.1186/s10020-018-0014-9

**Published:** 2018-04-04

**Authors:** Svetlana P. Chapoval

**Affiliations:** 1 Department of Microbiology and Immunology, University of Maryland School of Medicine, Baltimore, MD USA; 2 Center for Vascular and Inflammatory Diseases, University of Maryland School of Medicine, Baltimore, MD USA; 3 Program in Oncology at the Greenebaum Cancer Center, University of Maryland School of Medicine, Baltimore, MD USA; 4 SemaPlex LLC, Ellicott City, MD USA

**Keywords:** Costimulation, Immune response, Semaphorins, Molecular targets, Inflammation

## Abstract

Several neuronal guidance proteins, known as semaphorin molecules, function in the immune system. This dual tissue performance has led to them being defined as “neuroimmune semaphorins”. They have been shown to regulate T cell activation by serving as costimulatory molecules. Similar to classical costimulatory molecules, neuroimmune semaphorins are either constitutively or inducibly expressed on immune cells. In contrast to the classical costimulatory molecule function, the action of neuroimmune semaphorins requires the presence of two signals, the first one provided by TCR/MHC engagement, and the second one provided by B7/CD28 interaction. Thus, neuroimmune semaphorins serve as a “signal three” for immune cell activation and regulate the overall intensity of immune response. The current knowledge on their structures, multiple receptors, specific cell/tissue/organ expression, and distinct functions in different diseases are summarized and discussed in this review.

## Background

Molecular interaction between APC and T cells directs the development, sustainability, magnitude, and completeness of the immune response, and guides the direction of pathologic processes. Costimulatory molecules play a critical role in such interaction by regulating adaptive immunity and inflammation. According to the recent discoveries, two signals are necessary for optimal T cell activation (Pollizzi & Powell, 2014; Esensten et al., 2016). The first signal originates from the TCR interaction with an antigen (Ag)-derived peptide presented in the context of a specific MHC molecule. The second signal derives from the interaction of costimulatory molecules with their corresponding receptors. The signaling through TCR without costimulation leads to the development of T cell anergy. Recent discoveries of several novel costimulatory molecules and careful dissection of their in vitro and in vivo functions led to the conclusion that they provide either stimulatory or inhibitory signals to T cells depending on the receptor they interact with (Chen, 2004; Greenwald et al., 2005; Pollizzi & Powell, 2014; Esensten et al., 2016). With proper costimulation, three canonical signaling pathways, NF-kB, AP-1, and NFAT, are activated within T cells. This leads to the expression of many molecules, such as chemokines, cytokines, and other cell surface molecules, that promote T cell activation (Pollizzi & Powell, 2014). However, some molecules are already expressed on the surface of naïve immune cells. They actively participate in the initial stage of T cell response to Ag stimuli. The requirement of a “signal three” for an optimal T cell activation has been previously proposed (Reis e Sousa, 2006; Polizzi & Powell, 2014). Some studies considered certain cytokines fulfilling this role, such as IL-12 that directs naïve T cells into Th1 or CTL lymphocytes, or IL-6 that favors Th2 phenotype (Reis e Sousa, 2006). Other reports suggested that the cell surface molecules, such as Notch ligand (Reis e Sousa, 2006) and galectins (Méndez-Huergo et al., 2017), regulate the magnitude of T cell proliferation and cytokine response. In this review, we pinpoint neuroimmune semaphorins as the molecules that should be added to the list of the regulatory “signal three” for T cell response. They are either constitutively (Sema3A and Sema4A on APC, Sema4D, and Sema6D on T cells) or inducibly (Sema3A and Sema7A on activated T cells, Sema4A on Th1 cells, Sema4D on activated APC) expressed on immune cells and are tightly involved in the Ag-induced immune response. In this review, we also discuss in detail the discovery of neuroimmune semaphorins, their structures, receptors, tissue expression, expression regulation, and function in different diseases and experimental mouse models. Clear understanding of the multiple roles of neuroimmune semaphorins in physiologic and pathologic processes, and their further functional characterization as costimulatory molecules, will help to develop new strategies for manipulation of the immune response and immune-mediated diseases.

### Sema3A

#### Identification and structure

Sema3A, previously known as chick collapsin 1, SemD, or Sema III, was discovered in the 1990s. In the nervous system, it functions either as a repulsive agent for axonal outgrowth or an attractive agent for apical dendrite growth (Kolodkin et al., 1993; Luo et al., 1993; Puschel et al., 1995; Polleux et al., 2000). Sema3A is a glycoprotein with an Ig-like C2-type domain, a PSI (cysteine-rich module in extracellular portion) domain, and a Sema domain. It functions as a secreted homodimer consisting of 95 kDa monomers with neuropilin 1 (NRP-1) that serves as a ligand-binding receptor, whereas NRP-1-associated Plexin A1 functions as a signaling receptor (Takahashi et al., 1999; He & Tessier-Lavigne, 1997). The 95 kDa forms of Sema3A can further undergo a proteolytic cleavage forming the 65 kDa forms, which have decreased activity toward neurons (Adams et al., 1997; Klostermann et al., 1998).

#### Expression

Besides the nervous system, where it first was identified, Sema3A was detected in kidneys (Villegas & Tufro, 2002), oocytes (Assou et al., 2006), and adipose tissue (Giordano et al., 2003) (Table 1). An online source, The Human Protein Atlas, states that Sema3A can be found in the adrenal glands, heart muscle, respiratory epithelial cells in the nasopharynx, glandular cells in the duodenum, glandular cells in the prostate, and decidual cells in the placenta (Table 1). Recent studies have shown that Sema3A is expressed on and is secreted by immune (Vincent et al., 2005; Lepelletier et al., 2007) and endothelial (Serini et al., 2003; Kashiwagi et al., 2005; Toyofuku & Kikutani, 2007) cells.

**Table 1:** Semaphorin expression in tissues

Tissue	Semaphorin	References
Nervous system	Sema3A	Kolodkin et al., 1993; Luo et al., 1993; Puschel et al., 1995; Polleux et al., 2000
	Sema3E	Christensen et al., 1998
	Sema4D	Malik et al., 2015; Ito & Kumanogoh, 2016; Yang et al., 2016
	Sema7A	Pasterkamp et al., 2007
Immune system/cells	Sema3A	Vincent et al., 2005; Lepelletier et al., 2006; Lepelletier et al., 2007; Vadasz et al., 2015
	Sema4A	Kumanogoh et al., 2002b; Chakravarti et al., 2005; Kumanogoh et al., 2005; Kawamoto et al., 2011; Smith et al., 2011; Meda et al., 2012; Delgoffe et al., 2013; Zhou et al., 2014
	Sema4D	Elhabazi et al., 2001; Watanabe et al., 2001; Smith et al., 2011; Yang et al., 2016; Chapoval et al., 2017
	Sema6D	O'Connor et al., 2008
	Sema7A	Mine et al., 2000; Holmes et al., 2002; Pasterkamp et al., 2003; Czopik et al., 2006; Pasterkamp et al., 2007; van Rijn et al., 2016
Cancerous tissues/cells	Sema3A	Herman & Meadows, 2007; Tang et al., 2014; Jiang et al., 2015; Hu et al., 2016; Neufeld et al., 2016a; Wallerius et al., 2016; Yamada et al., 2016
	Sema3E	Christensen et al., 1998
	Sema4D	Liu et al., 2014; Neufeld et al., 2016a
	Sema6D	Chen et al., 2015
Kidneys	Sema3A	Villegas & Tufro, 2002
	Sema4D	The Human Protein Atlas
Oocytes	Sema3A	Assou et al., 2006
Adipose tissues	Sema3A	Giordano et al., 2003
Adrenal glands	Sema3A	The Human Protein Atlas
	Sema4D	The Human Protein Atlas
Heart muscle	Sema3A	The Human Protein Atlas
	Sema6D	The Human Protein Atlas
Duodenum	Sema3A	The Human Protein Atlas
Prostate	Sema3A	The Human Protein Atlas
	Sema6D	The Human Protein Atlas
Placenta	Sema3A	The Human Protein Atlas
	Sema4D	The Human Protein Atlas
	Sema7A	Yamada et al., 1999
Lungs	Sema3A	The Human Protein Atlas
	Sema3E	Christensen et al., 1998; Movassagh et al., 2017
	Sema7A	Roth et al., 2016
Endothelila cells	Sema3A	Serini et al., 2003; Kashiwagi et al., 2005; Toyofuku & Kikutani, 2007
Platelets	Sema3A	Kashiwagi et al., 2005
Eye (retina)	Sema3E	Sun et al., 2017
	Sema4A	Rice et al., 2004
Bone (osteobalsts/osteoclasts)	Sema3A	Hayashi et al., 2012; Liu et al., 2016
	Sema3E	Ryynänen et al., 2017
Colon	Sema4A	The Human Protein Atlas
	Sema6D	The Human Protein Atlas
	Sema7A	The Human Protein Atlas, Kang et al., 2012
Synovial fluids	Sema4A	Wang et al., 2015
Bone marrow	Sema4D	The Human Protein Atlas
Skeletal muscle	Sema4D	The Human Protein Atlas
	Sema7A	The Human Protein Atlas
Pancreas	Sema4D	The Human Protein Atlas
	Sema6D	The Human Protein Atlas
	Sema7A	The Human Protein Atlas
Salivary glands	Sema4D	The Human Protein Atlas
Esophagus	Sema4D	The Human Protein Atlas
Skin	Sema4D	The Human Protein Atlas
	Sema7A	The Human Protein Atlas
Female reproductive system	Sema4D	The Human Protein Atlas
	Sema6D	The Human Protein Atlas
	Sema7A	The Human Protein Atlas
Parathyroid glands	Sema6D	The Human Protein Atlas
Gallbladder	Sema6D	The Human Protein Atlas
Testis	Sema7A	Yamada et al., 1999

#### Function

**Fig. 1:**
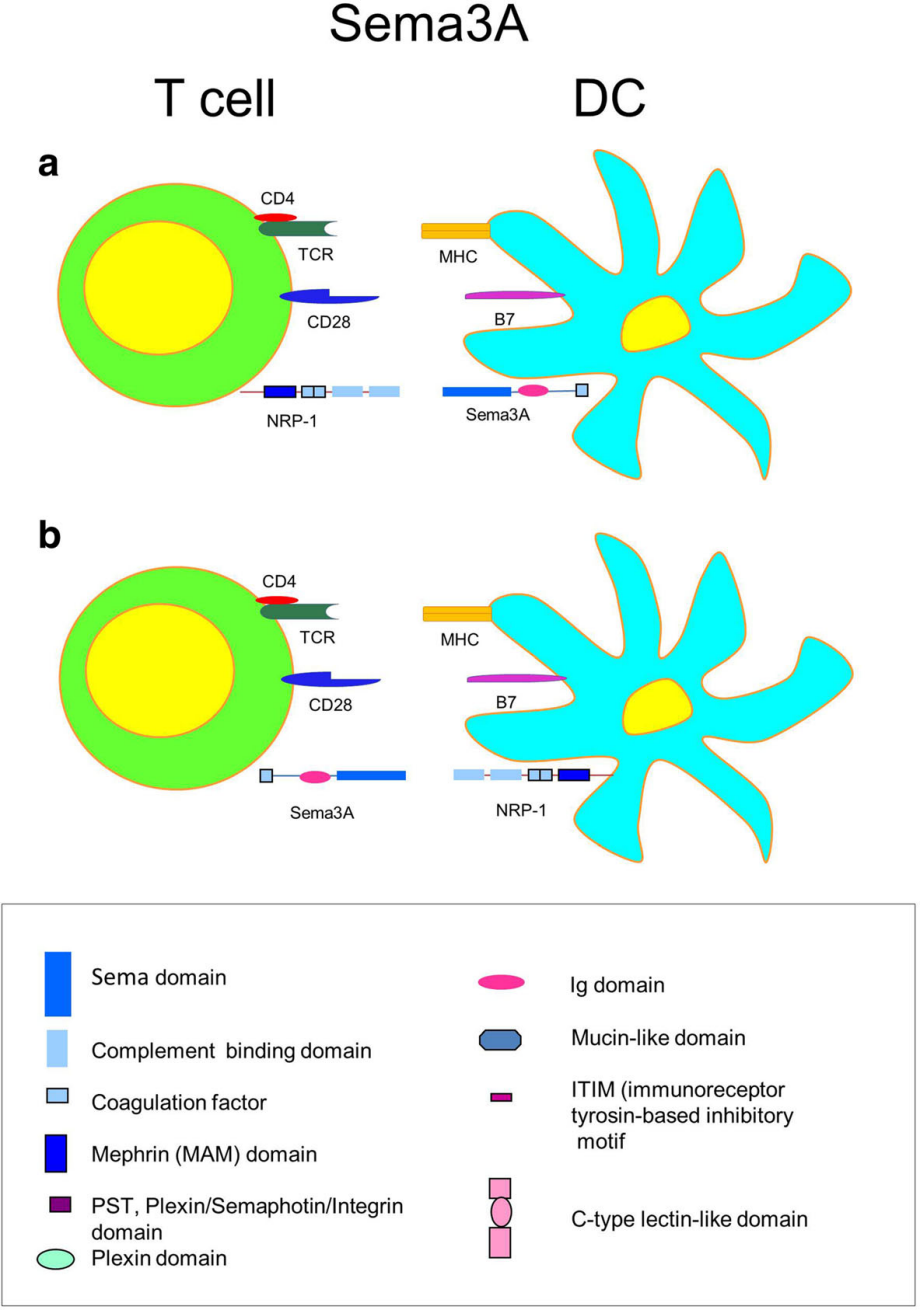
Sema3A in DC – T cell crosstalk: **a** Sema3A inhibits T cell activation. Low constitutive levels of Sema3A on DC are upregulated with cell activation. DC surface-expressed and soluble Sema3A inhibit T cell proliferation presumably acting through NRP-1. **b** Sema3A inhibits DC activation and chemotaxis. Inducible T cell-expressed and soluble Sema3A use NRP-1 and NRP-2 as ligand-binding receptors and NRP-associated Plexin A1 and A2 as signaling receptors to regulate DC activation and chemotaxis

In the immune system, Sema3A mRNA was found on anti-CD3/CD28-activated, but not on resting T cells (Lepelletier et al., 2006) (Fig. 1). Low levels of Sema3A mRNA were found in immature DC, which were significantly increased after TNF-α/IL-1β- or CD40L-induced cell maturation (Fig. 1). Interestingly, upon inflammatory cytokine stimulation of DC, Sema3A is upregulated as a 95 kDa isoform, whereas upon CD40L stimulation, it is further cleaved to a 65 kDa isoform. Thus, different inflammatory processes with variable cytokine milieu and cell-cell interactions can lead to the production of distinct Sema3A isoforms with potentially divergent pathologic results, what remains to be investigated. Activated T cells secrete Sema3A. Its secretion levels in DC-T cell cultures reached the maximum levels by 96 h and inhibited T cell activation. This suggests that Sema3A acts as an immune-regulatory molecule by limiting immune system over-activation (Fig. 1). The FACS studies with the use of either blocking Ab or antagonistic Sema3A peptide have shown that Sema3A binds to the NRP-1 receptor on T cells. Sema-3A blocked CD3/CD28-activated, but not PHA/IL-2-activated T cell proliferation. Conversely, anti-Sema-3A mAb increased T cell proliferation induced by anti-CD3/anti-CD28. Therefore, the work by Lepelletier and associates clearly demonstrates a novel inhibitory role of Sema3A in DC-T cell crosstalk.

In addition to the defined above regulation of T cell activation by Sema3A, Curreli and associates examined the role of class III semaphorin molecules in DC migration (Curreli et al., 2016). They have found that immature human DC (imDC) expressed NRP-1 and NRP-2. However, when NRP-2 expression increased with cell maturation, the NRP-1 expression declined. Similarly, we have previously demonstrated that VEGFR-2 is upregulated when mouse DC are activated, whereas VEGFR-1 expression decreased (Smith et al., 2011). It has been shown that NRP-1 enhances VEGFR-2 activity in the presence of VEGF, potentially as the result of a complex formation between VEGFR-2 and NRP-1 (Whitaker et al., 2001). In contrast, Sema3A inhibits VEGF-induced angiogenesis by competing with VEGF for binding to NRP-1 (Neufeld et al., 2002; Neufeld et al., 2002). Plexin-A1 and Plexin-A2 are also known to form complexes with NRP-1 and NRP-2 (Takahashi & Strittmatter, 2001). This formation is independent of semaphorin binding. Both imDCs and mature DC (mDCs) have been shown to express Plexin-A1 and -A3 mRNAs. Together with neuropilin and Sema3A, this suggests potential functional Sema/NRP/plexin signaling pathways within DC (Curreli et al., 2016). The authors also showed that Sema3A, -3C, and -3F all bind to human DC, with Sema3F binding predominantly to NRP-2. Receptor ligation by these semaphorin molecules induced cell actin filament\ reorganizations and increased transwell migration independently of the CCL19 chemokine. Therefore, several neuroimmune class III semaphorins, including Sema3A, display a chemotactic activity toward APC. This involves a complex interaction of different receptors and competing ligands, suggesting Sema3 modulation of immune response and inflammation.

Recent studies have shown Sema3A involvement in immune cell regulation (Vincent et al., 2005; Lepelletier et al., 2007), endothelial cell migration and proliferation (Serini et al., 2003; Toyofuku & Kikutani, 2007), and platelet function (Kashiwagi et al., 2005).

#### Disease relevance

Several reports have demonstrated the importance of Sema3A in cancer development and progression. However, the presented data have been controversial with regards to the positive or negative effects of Sema3A overexpression in certain types of cancer. The majority of published data supports an anti-tumoral role of Sema3A (Neufeld et al., 2016a), with some notable exceptions. On the one hand, it has been shown that an increased expression of Sema3A inhibits the cell invasiveness and adhesion of the prostate cancer (Herman & Meadows, 2007). On the other hand, it has been reported that in hepatocellular carcinoma, Sema3A promotes tumor development and expansion (Hu et al., 2016). Its tissue biopsy levels can serve as an independent predictor of the disease progression and survival time. In the latter study, the tissue immunohistochemistry has shown that in cancerous tissues, Sema3A is expressed on CD163+ M2 tumor-associated macrophages. Sema3A tissue level positively correlated with the tumor size, tumor numbers, tumor encapsulation, and tumor-node-metastasis staging. Another recent study has identified Sema3A as a necessary molecule for the maintenance of cancer stem-like cells in the lungs (Yamada et al., 2016). These cells’ eradication could lead to a prevention of tumor development or elimination of future metastasis. Thus, manipulating Sema3A expression could have many beneficial effects in tumor therapy.

It is important to note that the levels of Sema3A in different types of cancer have to be evaluated concomitantly with its receptors and the levels of competitive receptor-binding molecules, such as Sema4A (binds NRP-1), and known Plexin A1 ligands, namely - Sema3C, Sema3F, and Sema6D. The overexpression of a certain ligand while lacking sufficient numbers of available receptor(s) to interact could lead to different effects as compared to the ligand-receptor optimal expression and interaction levels. Similarly, the overexpression of different ligands for one receptor could lead to a competition for receptor-binding and, as a result, to different functional outcomes. Indeed, the complexity of the Sema3-NRP1-PlexinA1 system has been demonstrated in a recent study that defines the effects of class 3 semaphorins on DC (Curreli et al., 2016), which we discuss below.

Quantitative analysis of Sema3A expression in patients with systemic sclerosis (SSc) showed lower serum levels of Sema3A as compared to healthy controls and patients with diagnosed SLE (Rimar et al., 2015). Moreover, Sema3A expression on Treg (CD4 + CD25^high^) cells in SSc patients was also significantly lower as compared to that in control subjects. A clinical correlation study has shown that Sema3A serum level directly correlated with serum anti-Scl-70 (Scleroderma 70) Ab positivity and inversely correlated with disease onset (time of diagnosis) and low serum C4 levels. Similarly, Sema3A serum levels were lower in patients with Familial Mediterranean Fever (FMF) disease during attacks, in disease smoldering, or in remission as compared to the healthy controls (Rimar et al., 2016). The authors concluded that these findings suggest the potential role of Treg cells in FMF attack termination.

The effect and mechanisms of Sema3A action in rheumatoid arthritis (RA) has been examined in vitro using PBMC derived from RA patients and in vivo in a mouse model of collagen-induced arthritis (CIA) (Catalano, 2010). This study demonstrated that the major mechanism of inhibitory Sema3A effect in RA is its suppression of activated CD4+ T cells. The CD4+ T cells from RA patients were found to express two Sema3A receptors, NRP-1 and Plexin A1, but had lost Sema3A expression, and were unable to upregulate Sema3A even under T cell stimulating conditions. In vivo, the overexpression of Sema3A induced IL-10 production mainly in CD4 + NRP-1+ T cells, and reduced IFN-γ and IL-17, what was sufficient to prevent CIA and to arrest disease progression. Therefore, an upregulation of Sema3A inhibited a proinflammatory response and attenuated a CIA development what suggests its therapeutic efficacy in RA and other autoimmune diseases.

Sema3A also plays a role in bone remodeling by stimulating osteoblastogenesis but inhibiting osteoclastogenesis (Hayashi et al., 2012; Liu et al., 2016). It is well known that bone structural balance is regulated by osteoblast-mediated bone formation and osteoclast-mediated bone resorption. Sema3A was found to be expressed by osteoblasts but not by osteoclasts and to inhibit osteoclastogenesis via NRP-1 (Hayashi et al., 2012). This pathway activation led to a suppression of the osteoclastic genes Ctsk, Acp5 and Nfatc1 expression. Moreover, Sema3A^−/−^ and NRP-1^Sema-^ mice demonstrated a severe bone mass loss associated with a canonical Wnt signaling pathway dysregulation. When exogenous Sema3A was reintroduced to Sema3A^−/−^ mice, an increase in a bone formation and accelerated bone regeneration effect were observed what suggests the osteoprotective effects of this neuroimmune semaphorin. Liu and associates (Liu et al., 2016) used the in vitro cultures of bone marrow mesenchymal stem cells for osteoblasts and osteoclastic RAW264.7cells. When analyzing the effect of wedelolactone, a compound isolated from Ecliptae herba, used in the treatment of bone-mediated diseases such as osteoporosis, the authors have found that wedelolactone induced Sema3A in a dose-dependent manner. Wedelolactone was found to promote osteoblastogenesis through Sema3A by inducing the formation of the Sema3A-PlexinA1-NRP-1 complexes and b-catenin activation. In osteoclastic cells, wedelolactone inhibited osteoclastogenesis through sequestration of the PlexinA1-DAP12 complex, induced the formation of PlexinA1-NRP-1 complexes, and suppressed PLCg2 (phospholipase C which acts downstream of the PlexinA1/NRP-1/DAP12pathway) activation. These data clearly demonstrated an important role of Sema3A as a regulatory molecule in bone homeostasis and bone loss. Indeed, the spontaneous bone loss was detected in Sema3A^−/−^ mice (Hayashi et al., 2012; Fukuda et al., 2013).

As we outlined above, Sema3A’s function in cancer is still unclear and needs to be defined in vitro using cancer cell lines and in vivo using mice lacking Sema3A and/or corresponding individual receptor expression generated by different techniques that are currently available. Nevertheless, one of the mechanisms of tumor suppression by Sema3A has been recently demonstrated by Wallerius and associates (Wallerius et al., 2016). Their study showed that tumor cell-derived Sema3A inhibited proliferation and expansion of pro-tumor M2 macrophages, but increased the number of anti-tumor M1 macrophages. Moreover, it has been shown that Sema3A expression inversely correlated with an increasing degree of malignancy in different types of cancer, including ovarian, breast, gastric, and non-small cell lung cancer, when compared with corresponding normal tissue (Jiang et al., 2015; Tang et al., 2014; Zhou et al., 2014). Thus, Sema3A acts as a tumor suppressor, but its suppressor activity needs to be further explored and used in a potential therapeutic anti-tumor intervention.

### Sema3E

#### Identification and structure

Sema3E (originally termed M-SemaH) was first identified in the metastatic cell lines using a differential display technique which allowed to identify 2 splice variants encoding the same 775 a.a. protein (Christensen et al., 1998). The protein consists of a putative signaling sequence in NH- terminus followed by a large semaphorin domain, a c2 immunoglobulin-like domain at the amino acids 595–659, an approximately 20 residues serving as a transmembrane domain, and positively charged residues in the COOH-terminus (Christensen et al., 1998). Sema3E contains 13 conserved cysteine residues, and 3 potential A’-glycosylation sites. The amino acid sequence of Sema3E was found to be 82% identical to the reported partial sequence of chick collapsin 5 and 44–48% to all other members of the subclass III of the family (Christensen et al., 1998). Also, the AU-rich motif (AUUUA) conferring protein instability has been defined.

#### Expression

The Northern blot analysis of the adult mouse tissues revealed the Sema3E RNA expression in the brain and lungs but not in the heart, liver, skeletal muscle, kidney, spleen, or testis (Christensen et al., 1998) (Table 1). Within the human lung tissues, Sema3E was readily detectable on bronchial epithelial cells (Movassagh et al., 2017a) whereas one of its receptors, Plexin D1 was detected on airway smooth muscle and endothelial cells (Movassagh et al., 2014; Gu et al., 2004). Two recent studies found Sema3E on osteoblasts (Ryynänen et al., 2017) and retinal ganglion (Sun et al., 2017) where it regulates bone homeostasis and retinal angiogenesis, correspondingly.

#### Function

**Fig. 2:**
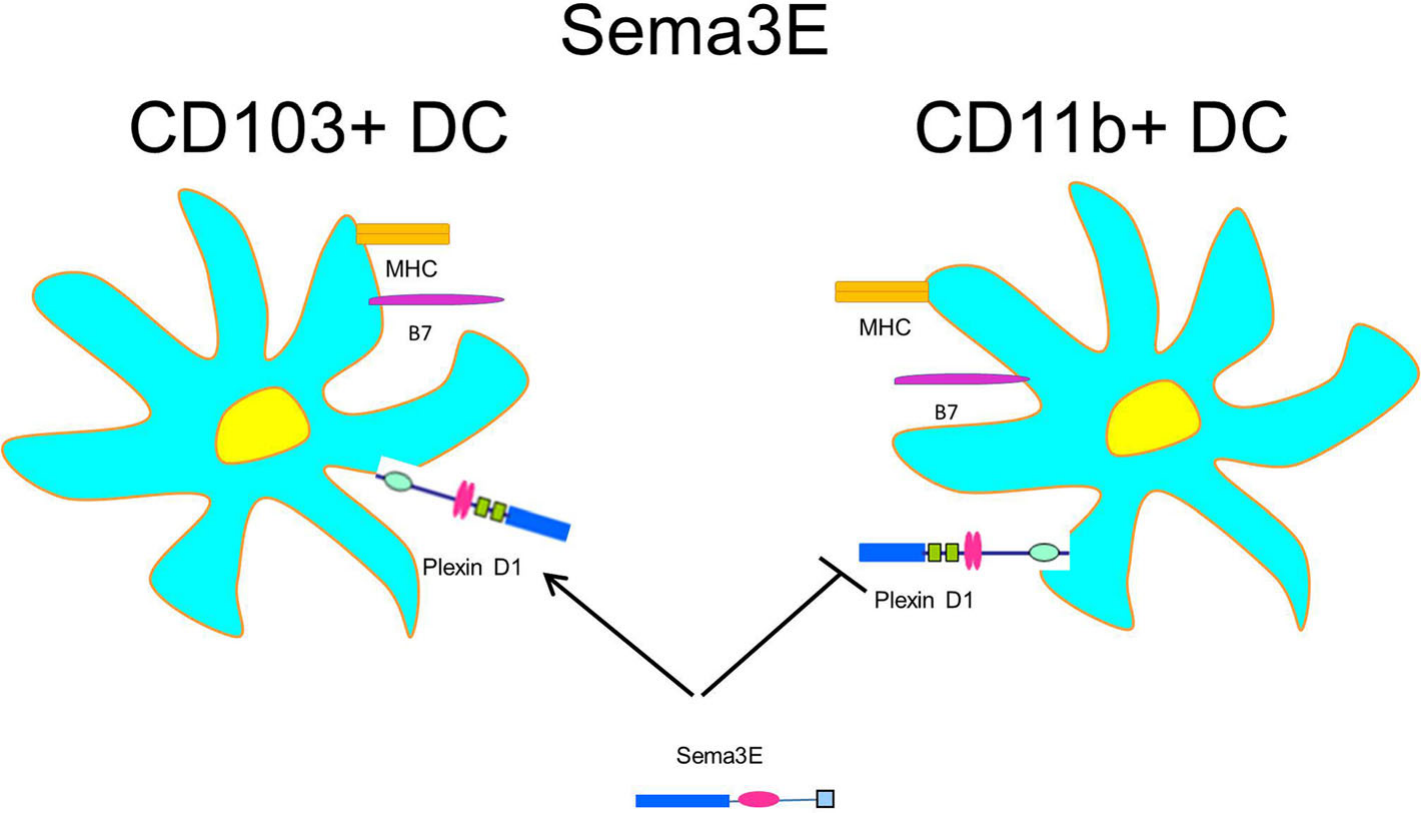
Sema3E regulates DC subsets. Higher numbers of CD11b + DC and lower numbers of CD103+ DC were detected in the lungs of Sema3E^−/−^ mice at the steady-state condition and after allergen sensitization. The DC receptor involved in such Sema3E action is Plexin D1

Several recent reports have defined Sema3E involvement in cell migration (Movassagh et al., 2014; Movassagh et al., 2017b; Mecollari et al., 2014; Mishra et al., 2015; Nasarre et al., 2014), proliferation (Nasarre et al., 2014), and angiogenesis (Gu et al., 2004). The latter study has identified Plexin D1 as a receptor for Sema3E on endothelial cells of blood vessels. Sema3E was found to be highly expressed in developing somites and to act as a repulsive cue for endothelial cells of adjacent intersomitic vessels. Sema3E-Plexin D1 signaling did not require neuropilins. Moreover, Sema3E or Plexin D1 deficiency led to a disrupted vascular arrangement what suggests that Sema3E-Plexin D1 signaling pathways control the vascular patterning (Gu et al., 2004). This signaling pathway is also involved in DC subset regulation (Fig. 2).

#### Disease relevance

Tumor Sema3E expression has been reported to positively correlate with a disease progression (Christensen et al., 1998). Sema3E has been found to be expressed on all myeloid cell lines but only on two out of six tested non-metastatic cell lines.

The work by Movassagh and associates (Movassagh et al., 2017) defined the effect of Sema3E deficiency in experimental mouse model of asthma. Such deficiency resulted in a substantial increase of many components of asthmatic response. This increase involved AHR, mucus secretion, collagen deposition, and Th2/Th17 lung inflammation. All these features of asthmatic response were significantly downregulated when recombinant Sema3E was administered to the allergen-sensitized mice intranasally (Movassagh et al., 2017). A higher frequency of CD11b + pulmonary DC, a Th2-promoting subtype of DC, was observed in Sema3E^−/−^ mice in both, the steady-state and allergen-sensitized conditions as compared to WT control animals (Fig. 2). When adoptively transferred to naïve mice, these Sema3E^−/−^ CD11b + DC were able to induce the highest allergic lung inflammatory response especially when the DC recipients were Sema3E^−/−^ mice. While examining the generated bone marrow chimeric mice, the authors defined the contribution of Sema3E on bone marrow-derived inflammatory cells in allergen-induced lung pathology. This work aligns with their previous study demonstrating Sema3E-mediated inhibition of human ASM cell proliferation and migration and defining the signaling pathways involved in such effect (Movassagh et al., 2014). Moreover, their recent study clearly demonstrated a suppressed Sema3E expression in human severe asthma using bronchial biopsy and lung tissue histology speciments (Movassagh et al., 2017a). These data suggest that Sema3E plays an important regulatory role in allergic asthma. Sema3E-mediated cellular effects involve, first of all, pulmonary DC and bronchial epithelial cells. Targeting this molecule could be a novel approach to treat allergic asthma.

### Sema4A

#### Identification and structure

In 1995, Puschel and associates (Puschel et al., 1995) discovered Sema4A (previously known as RP35, SEMB, SEMAB, and CORD10) and reported its sequence. They screened several different cDNA libraries (a mouse brain ZZAP cDNA library, a mouse E12 embryo ZT_AP cDNA library, a mouse E12 spinal cord ZZAPExpress cDNA library, and a mouse E12 embryo Z7_.APExpress cDNA library), using primers derived from conserved motifs of the semaphorin domain. Sequences sharing homology with the semaphorin/collapsin genes were amplified by PCR. With this procedure, cDNAs for five different semaphorins were isolated and initially termed as Sem A-Sere E molecules. This led to the characterization of the expression patterns of these newly discovered semaphorin molecules in the neural and mesodermal tissues and to the investigation of their effects on the neurite growth. The Sema4A molecule is a 761 aa long glycoprotein of 150 kDa molecular weight with an NH2-terminal 32 aa signal peptide, a Sema domain and an Ig domain of the C2 type (both 651 aa), a hydrophobic 21 aa transmembrane region, and a 57 aa cytoplasmic tail (Swissprot Accession # Q9H3S1). Its functions are the most complicated, diverse, and least studied. Sema4A has six known receptors. The discrepancy between published in vitro and in vivo research results suggests that an unknown receptor(s) for this unique semaphorin might be present. Sema4A exists in both membrane-bound and soluble forms (Smith et al., 2011; Toyofuku et al., 2007). On the cell surface, it is expressed as a monomer and a dimer (Toyofuku et al., 2007). There are six reported splice variants of Sema4A: 723, 629, 370, 321, 236, and 220 aa long (Schulz et al., 2014). According to Schulz and associates, “five of them lack the N-terminus and/or portions of the Sema domain, and three of them lack the transmembrane and cytoplasmic domains in the C-terminus”.

#### Expression

Sema4A was found to be expressed in several tissues including brain, lung, and lymphoid organs (Table 1). In lymphoid tissues, Sema4A is expressed on immature APC under steady state conditions (Kumanogoh et al., 2002a). Although its expression was not detected on resting T cells, T cell activation induced Sema4A expression on cell surface, although to a lesser extent than that observed on APC. Further T cell examination has shown the restriction of Sema4A expression to Th1 cells (Kumanogoh et al., 2002a). Sema4A has six functional receptors, namely Plexin -D1, -B1, -B2 and -B3 (Malik et al., 2015; Ito & Kumanogoh, 2016), Tim-2 (Kumanogoh et al., 2002a), and NRP-1 (Delgoffe et al., 2013). Immune cells in the lungs (DC, macrophages, T cells, and B cells) were found to express low levels of Sema4A, which was potentiated by VEGF exposure (Smith et al., 2011). Lung exposure to allergen and local VEGF expression in transgenic mice induced Tim2+ cell influx into the lung tissue. Interestingly and unexpectedly, Tim-2 positivity was detected on the subsets of macrophages and granulocytes besides lymphocytes (Smith et al., 2011). This suggests that Tim-2 is not a definite marker for the late differentiated Th2 cells (Chakravarti et al., 2005) or activated B cells (Kawamoto et al., 2011) as it can be expressed by other activated cells of non-lymphoid origin under inflammatory conditions, at least in the lung tissue.

#### Function

**Fig. 3:**
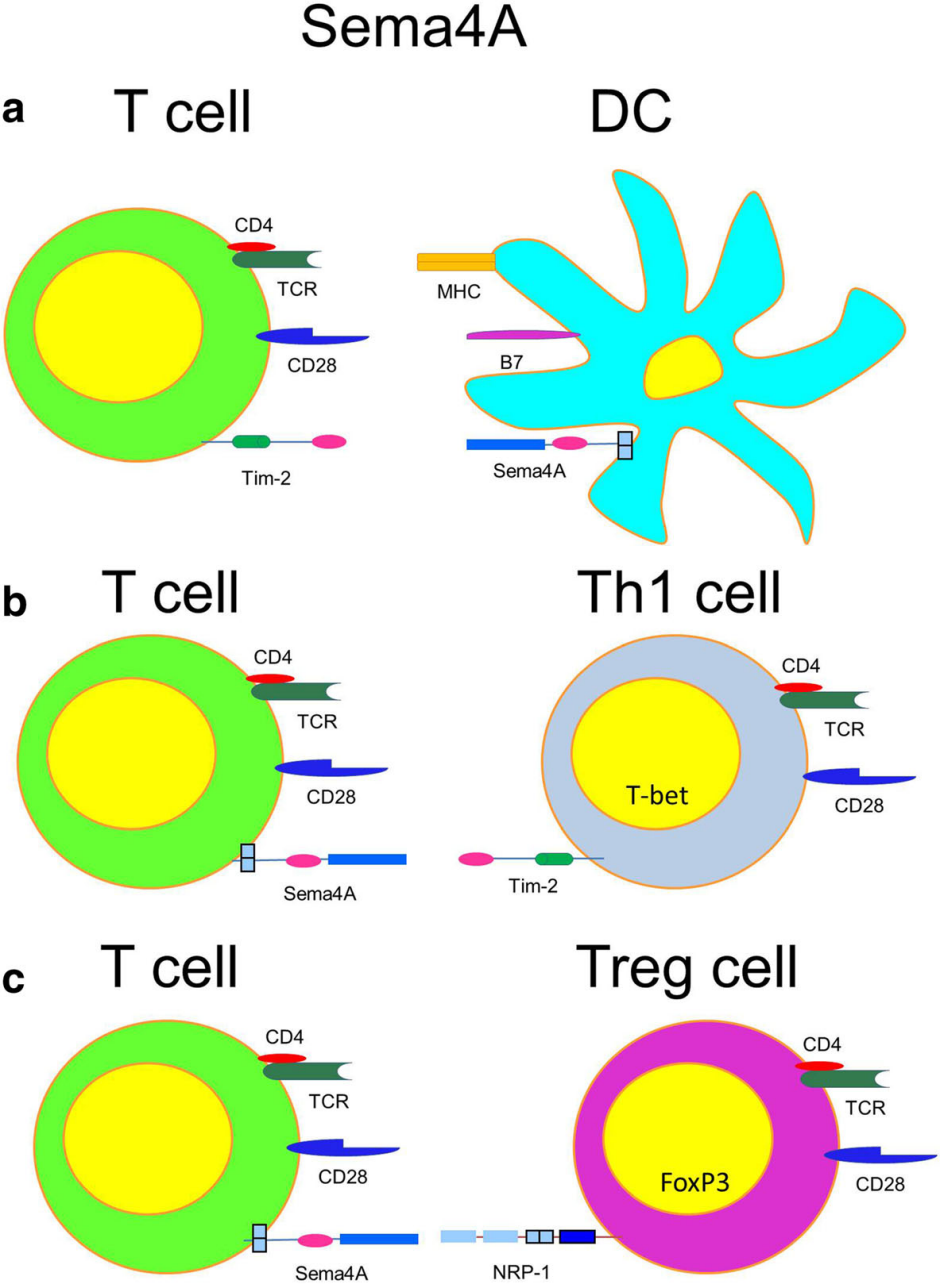
Different roles of Sema4A in the immune response. **a** Sema4A-Tim2 pathway costimulates T cells. Sema4A on DC directly binds Tim-2 on T cells. This leads to optimal T cell activation, proliferation and cytokine production. **b** Sema4A in T-T cell interaction. Sema4A ligation of Tim-2 expressed on Th1 cells optimizes Th1 effector immune response. **c** Sema4A in T-Treg cell interaction. Expressed on conventional T cells, Sema4A ligates NRP-1 on the surface of T reg cells. This interaction induces a complex of signaling events in T reg cells directed to promote their stability and function

As Sema4A is constitutively expressed on DC, its role in DC activation and function was extensively studied in vitro and in vivo by Hitoshi Kikutani’s group (Kumanogoh et al., 2005). They were the first to link the selected semaphorins to the immune system. They have reported that Sema4A^−/−^ DC mature normally and respond to polyclonal stimulation (CD40 and LPS) as efficiently as WT DC; however, they were unable to induce an optimal T cell stimulation in a mix lymphocyte reaction (MLR) (Kumanogoh et al., 2005). The addition of a recombinant Sema4A to the cultures of the in vitro TCR- and CD28-stimulated T cells led to a significant upregulation of their activation and cytokine production (Kumanogoh et al., 2002a). This suggests that Sema4A acts as a costimulatory molecule for T cell activation (Fig. 3). In vivo, Sema4A^−/−^ mice showed the substantially lower T cell responses to several Ags (KLH, myelin oligodendrocyte glycoprotein (MOG) peptide, and nematode) (Kumanogoh et al., 2005). Thus, Sema4A seems to be a necessary molecule for optimal T cell stimulation and response to Ag. However, in contrast to all of the above, an increased allergic Th2-driven asthmatic response was observed in Sema4A^−/−^ mice after allergen exposure (Nkyimbeng-Takwi et al., 2012; Morihana et al., 2013). Treatment of these mice with Th2 response-inducing *Nippostrongulus brasiliencis* showed an enhanced inflammatory response as compared to WT mice (Kumanogoh et al., 2005). Thus, Sema4A on DC is involved in Ag-specific T cell activation (Fig. 3) and stimulates both Th1 and Th2 responses in vitro. In vivo, however, Sema4A acts as a suppressor of a Th2 phenotype and as a stimulator of a Th1 phenotype. It has not been determined yet if such discrepancy between the in vitro and in vivo results can be explained, in part, by a different receptor or a particular combination of receptors involved in Sema4A’s actions in different tissues and organs. The presence and signaling of distinct Sema4A receptors on different immune cells in vivo can also influence the outcome of its action in the immune response to Ag. For some time, it was believed that the only functional receptor for Sema4A on T cells was Tim-2 (Kumanogoh et al., 2002a). However, a more recent study has shown that Sema4A is functionally engaged with NRP-1 on Treg cells; this engagement is necessary for Treg cell stability and function at the sites of inflammation (Delgoffe et al., 2013) (Fig. 3). We and others have shown that DC express Sema4A receptors Plexins B1, B2, and D1 (Smith et al., 2011; Holl et al., 2012), which can also modify the immune response to Ag by modulating DC activation. Indeed, the absence of either Plexin B2 or Plexin D1 molecule on DC stimulated with anti-CD40 or LPS led to an upregulation of IL-12/IL-23p40 production (Holl et al., 2012). Although both plexins showed various levels of expression on DC, they also both negatively regulate IL-12/IL-23p40. This suggests a possible crosstalk between these two pathways. Sema4A is inducibly expressed on T cells of the Th1 phenotype (Kumanogoh et al., 2005), which adds to the complexity of how the Sema4A system works under inflammatory conditions. Interestingly, it has been shown that Sema4A on DC and Th1 cells have distinct functions in the T cell-mediated immune response (Kumanogoh et al., 2005). When T cells were cultured in Th1-polarizing conditions in the presence of IL-12 and anti-IL-4 Ab, they were induced to express high levels of Sema4A. When T cells were cultured in Th2-polarizing conditions, their Sema4A expression levels were low and transient (Kumanogoh et al., 2005). Sema4A^−/−^ T cells differentiated normally into Th2 cells, but failed to differentiate into Th1 cells and to produce IFN-γ due to a lower expression of the IL-12Rβ2 chain on the cell surface and lower intracellular t-bet levels. Both molecules are necessary for Th1 cell generation (Szabo et al., 2000) (Fig. 3). Thus, under chronic inflammatory conditions underlying many diseases, Sema4A-expressing Th1 and Treg cells can significantly interfere and modify the outcome of the immune response.

Under different conditions and on different cells, soluble Sema4A acts either as a chemoattractant or chemorepellent. For example, Sema4A has been shown to increase macrophage migration that operate through its Plexin D1 receptor (Meda et al., 2012) and to decrease endothelial cell (EC) migration as well as angiogenesis acting through the same functional receptor (Toyofuku et al., 2007). In the first scenario, Sema4A promotes angiogenesis as Sema4A-stimulated macrophages enhance VEGF-A expression, which, in turn, further increases macrophage activation and enhances VEGFR2 and PI3K/serine/threonine kinase Akt pathway expression by EC. The authors suggest that Sema4A chemotactic activity toward macrophages may be direct although it has not been tested in this study. This conclusion is based on the observation that macrophage exposure to Sema4A did not significantly change the expression levels of any inflammatory chemokine and only slightly increased IL-8 content. On the other hand, the direct effect of soluble Sema4A on EC was the opposite to its effect on macrophages, as the VEGF165-potentiated migration of HUVECs was suppressed by Sema4A-Fc (Toyofuku et al., 2007). In contrast to Sema4A’s effect on macrophages, where it stimulated VEGF-A production, its direct effect on EC was characterized by a suppression of VEGF-mediated activation of Rac and both Akt and Erk1/2. Thus, distinct effects of Sema4A on different cell types, even if using the same receptor, should be taken into account when designing a therapeutic strategy to either potentiate (for allergic asthma) or inhibit (for rheumatoid arthritis) its activity. It is important to mention that only certain agents can induce Sema4A expression in macrophages (Meda et al., 2012). The expression was strongly stimulated by LPS, poly:IC, and to a lesser extent by CpG, but not by flagellin, R848, and PAM3Cys. Sema4A expression was higher on unstimulated and thioglycollate-induced Ly6Chigh, but not on Ly6Clow peripheral blood monocyte subsets (Meda et al., 2012). The authors therefore suggested that Sema4A belongs to a specific signature for circulating inflammatory monocytes that migrate to the sites of tissue damage.

#### Disease relevance

Soluble Sema4A levels were found to be significantly lower in patients with Crohn’s disease and ulcerative colitis as compared to control subjects (Vadasz et al., 2015). At the same time, increased expression of membrane-bound Sema4A was observed in bowel biopsies of these patients; this suggests that there is an upregulation of intestinal Sema4A expression without its notable shedding into circulation, but rather into local areas. The significance of such observations for pathogenesis and prognosis of Crohn’s disease needs to be investigated further.

Sema4A also plays a critical role in retina formation. At the age of 14 weeks, Sema4A^−/−^ mice have been shown to develop severe retinal degeneration with attenuated retinal vessels and depigmentation (Rice et al., 2004). Detailed examination of the underlying pathologic mechanism of retinal degeneration has shown that Sema4A deficiency led to a disruption of the rod and cone photoreceptor function.

The utility of Sema4A for biomarker discoveries has been demonstrated in a recent study by Wang and associates (Wang et al., 2015). This study suggests that Sema4A might serve as a diagnostic and prognostic marker for the initiation, progression, and therapeutic intervention of rheumatoid arthritis. The authors detected the overexpression of a soluble Sema4A molecule in synovial fluids and sera of RA patients. Moreover, the levels of secreted Sema4A positively correlated with the disease activity score. The invasive ability of RA synovial fibroblasts (RASF) was potentiated by recombinant human Sema4A (rhSema4A) and blocked with Sema4A small interfering RNA (siRNA), thus showing a pro-invasion activity of Sema4A, which induces MMP-3 and MMP-9 expression. Furthermore, this study demonstrated the positive stimulatory Sema4A-nuclear factor-kappa-B (NF-κB) loop as the siRNA-mediated silencing of either p50 or p65 led to a downregulation of Sema4A expression in RASFs and THP-1 cells, whereas rhSema4A treatment upregulated NF-κB phosphorylation in RASFs. Also, rhSema4A-dependent IL-6 production in RASFs was attenuated by a “specific NF-κB inhibitor, inhibited by silencing Plexin B1, and partially inhibited by silencing Plexin D1 and Tim-2”. These data demonstrate that all three Sema4A receptors are involved in the RA pathogenesis; however, the strength of an individual receptor’s involvement differs. Moreover, the LPS- induced release of other pro-inflammatory cytokines, namely TNF-α and IL-1β, in Th1 cell line was significantly promoted by rhSema4A. Importantly, the above-mentioned cytokines are critical players in the RA pathogenesis, and their suppression had stalled disease progression and significantly improved disease activity scores (Edwards, 2005).

### Sema4D

#### Identification and structure

Sema4D, also known as Cluster of Differentiation 100 (CD100), was the first semaphorin which expression and function was defined in lymphoid cells (Hall et al., 1996; Furuyama et al., 1996). Later, its function as an axon guidance molecule in adult CNS was shown (Swiercz et al., 2002). Several studies pointed to its critical regulatory role in the immune system (Hall et al., 1996; Bougeret et al., 1992; Herold et al., 1995; Adams et al., 1996), which we discuss below. Sema4D consists of an NH2-terminal signal peptide, a sema domain, an Ig domain of the C2 type, a hydrophobic transmembrane region, and a cytoplasmic tail (Hall et al., 1996; Furuyama et al., 1996). The molecule’s crystal modelling demonstrates the presence of a conserved seven-blade β-propeller structure (Love et al., 2003) which is the structure of a conserved sema domain and is shared by all semaphorin family members. There is an 88% amino acid identity between human and murine Sema4D homologs (Furuyama et al., 1996). Sema4D exists in both, membrane-bound and soluble forms, which are both biologically active (Elhabazi et al., 2001; Zhu et al., 2007).

#### Expression

According to the Human Protein Atlas (http://www.proteinatlas.org), Sema4D expression was found in the adrenal gland, on hematopoietic cells in bone marrow, on skeletal muscle myocytes, in the pancreas and salivary glands (glandular cells), the esophagus, the kidneys, the cervix, the uterus, the ovaries, the placenta, and skin (Table 1). The same website lists the predicted 13 splice variants of a Sema4D protein. Another website, the GeneCards/Human gene database (http://www.genecards.org/cgi-bin/carddisp.pl?gene=SEMA4D) lists a limited number of cells and tissues for Sema4D expression. Sema4D was found on immune cells, the frontal cortex of brain, the spinal cord, the retina, the heart, the oral epithelium, the kidneys, the spleen, the pancreas, the ovaries, and the testis. A recent study has also shown its constitutive expression on human eosinophils (Hiraguchi et al., 2015).

As for the immune system, several research groups have demonstrated Sema4D expression on resting T cells and its upregulation upon T cell activation (Smith et al., 2011; Hall et al., 1996; Delaire et al., 1998; Kumanogoh et al., 2002b; Chabbert-de Ponnat et al., 2005). Previously, it has been reported that its receptor on immune cells, for example DC, is CD72 (Kumanogoh et al., 2000) whereas Plexin B1 serves as a Sema4D receptor in non-lymphoid tissues (Kumanogoh et al., 2002b; Tamagnone et al., 1999). More recent studies have shown that mouse DC express Plexin B1 (Smith et al., 2011; Holl et al., 2012), which added to the complexity of Sema4D-initiated pathways in immune cell activation and function. Defective in vitro and in vivo priming of Sema4D^−/−^ T cells was partially rescued by the in vivo rSema4D administration (Shi et al., 2000) what suggests that Sema4D could also serve as a ligand for T cell activation. It was proposed that activated T cells might express CD72 which could be involved in Ag-specific T cell response (Shi et al., 2000). Indeed, we have demonstrated that the small fractions of lung CD4+ and CD8+ T cells express CD72 and that the levels of expression are potentiated by allergen exposure for CD4 + T cells (Smith et al., 2011). Thus, activated T cells in different tissues under certain inflammatory conditions could potentially display an autocrine and/or paracrine Sema4D-CD72 pathway signaling effects. Further detailed analysis of this “reverse” pathway in T cell priming is required to clarify the role of CD72 on T cells.

#### Function

**Fig. 4:**
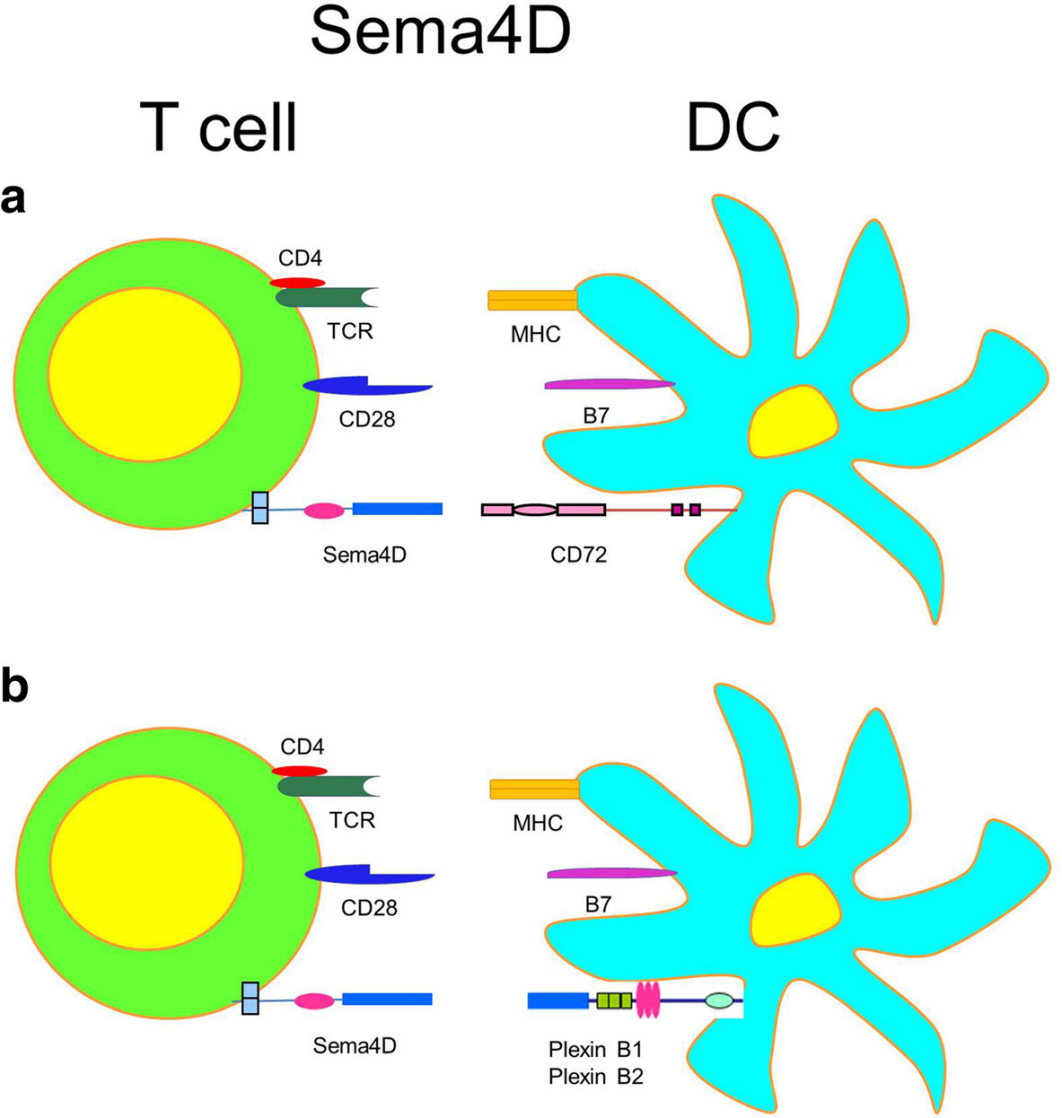
Distinct receptor-dependent effects of T cell-expressed Sema4D on DC functions. **a** Sema4D costimulates T cells. Sema4D serves as an indirect costimulatory molecule for T cell activation. Sema4D on T cells stimulates DC to accelerate their activation and maturation. Stimulated DC, in their turn, enhance T cell activation. The main receptor for such Sema4D action is believed to be CD72. **b** Sema4D costimulates DC. T cell-expressing and soluble Sema4D ligation of DC-expressing Plexin B1 and B2 receptors stimulates DC proinflammatory cytokine production and migration

The functional importance of Sema4D for T cell activation was demonstrated in vitro using molecule-deficient and -proficient immune cell co-cultures with Ag. This showed that Sema4D on T cells stimulated DC to promote their activation and maturation, and these stimulated DC, in turn, enhanced T cell activation (Kumanogoh et al., 2002b) (Fig. 4). The main receptor for this Sema4D action was believed to be CD72. Thus, Sema4D serves as an indirect costimulatory molecule for T cells.

Generation of Sema4D^−/−^ mice has allowed studying its function in vivo. In vivo, Ag-primed T cells from Sema4D^−/−^ mice showed lower proliferation and cytokine production after in vitro Ag re-stimulation (Kumanogoh et al., 2002b; Shi et al., 2000; Shanks et al., 2013). Such impaired T cell priming was partially rescued by the in vivo rSema4D administration (Shi et al., 2000), what suggests that Sema4D can potentially serve as a direct costimulatory molecule for T cells. The critical importance of this pathway for an optimal T cell proliferation was recently tested using several commercially available anti-Sema4D Abs which have been shown to effectively inhibit T cell proliferation (Jiang et al., 2017). The observed anti-Sema4D Ab inhibitory effects were comparable to the use of a soluble blocking anti-CD72 Ab. Furthermore, when CD72-Fc was introduced to the cultures of polyclonally (anti-CD3/anti-CD28 coated beads) stimulated PBMC or purified T cells, the inhibitory effects of the above mentioned Abs were eliminated. Sema4D^−/−^ mice were not able to develop EAE after the MOG65 peptide immunization as compared to WT mice (Kumanogoh et al., 2002b). In contrast, transgenic Sema4D overexpressing mice demonstrated enhanced T cell responses (Watanabe et al., 2001). Similar to OVA-treated WT mice, Sema4D^−/−^ mice have shown reduced allergic lung inflammation, which was associated with lower Th2 and Th17A cytokine levels, and decreased in vitro Ag recall T cell proliferation, but did not affect lung AHR to methacholine challenges (Shanks et al., 2013). Overall, all these above mentioned studies showed the importance of Sema4D for T cell activation, priming and differentiation, and T cell-dependent Ag-mediated disease manifestation.

#### Disease relevance

Most studies concerning the role of Sema4D in cancer indicate a pro-tumoral activity of this semaphorin acting on the diverse components of cancer development and progression, including cancer cells, blood vessels, infiltrating macrophages, etc. (Ch'ng & Kumanogoh, 2010; Neufeld et al., 2016b; Gurrapu et al., 2016; Chapoval et al., 2017). It has been shown that reduced Sema4D tissue expression in breast cancer patients was associated with a lower degree of disease-free survival and local disease recurrence (Malik et al., 2015). In this particular study, Sema4D expression was examined together with its plexin family (Plexin – B1, − B2, and – B3) receptor expression. All markers were found to be downregulated in recurrent tumors as compared to the breast tissues of disease-free patients. This contrasted with a recently published study defining the Sema4D and Plexin B1 expression levels in colorectal cancer, where increased expression of these molecules was found to be associated with a poorer prognosis (Ikeya et al., 2016). The Sema4D-Plexin B1 expression was found to be significantly related to tumor’s stage, depth of tumor invasion, lymph node metastasis, lymphatic invasion, and venous invasion, but unrelated to the patient’s age and gender. These findings are in accord with studies performed in cervical cancer patients (Liu et al., 2014). The increased Sema4D expression in these patients was associated with increased VEGF-C/VEGF-D levels, the presence of lymphatic invasion, the occurrence of lymph node metastasis, the International Federation of Gynecology and Obstetrics (FIGO) disease stage, and poor survival prognosis. Similarly, in non-small cell lung carcinomas, Sema4D expression serves as an early predictor of a poor prognosis as its overexpression is associated with a poor pTNM (Classification of Malignant Tumours) staging and occurrence of lymph node metastasis. As a result, the downregulation of Sema4D and its receptor expression can be an attractive immunotherapeutic approach for the treatment of many types of cancer. Moreover, anti-Sema4D Ab delayed tumor growth and prolonged survival by 29% in a murine colon cancer model (Evans et al., 2015) and Sema4D silencing by RNA interference reduced the breast cancer related bone metastasis (Yang et al., 2016). A combination of anti-SEMA4D Ab with CTLA-4 Ab had a synergistic effect in complete tumor rejection, was more effective in tumor inhibition than anti-CTLA-4 Ab and anti-PD-1 Ab combination, and more significantly prolonged survival (Evans et al., 2015). The authors concluded that SEMA4D inhibition represents a new therapeutic strategy aimed at inhibiting tumor progression by promoting functional immune cell infiltration into the tumor microenvironment. Recently reported.

Clinical studies in patients with rheumatoid arthritis (RA) have also demonstrated the importance of Sema4D-mediated pathways in disease pathogenesis, even when Ag is not clearly defined (Yoshida et al., 2015). The elevated levels of soluble Sema4D in both serum and synovial fluids, which directly correlated with disease activity markers, were detected in RA patients. The in vitro recombinant Sema4D exposure of synovial cells led to the pro-inflammatory cytokine production. The in vivo blocking of Sema4D with Ab had a beneficial therapeutic effect in the experimental mouse model of CIA, lowering the disease score and pro-inflammatory cytokine production (Yoshida et al., 2015). The authors suggest that a positive feedback loop involving sSema4D/IL-6 and TNFα/ADAMTS-4 participates in RA pathogenesis and that anti-Sema4D Ab or other specifically Sema4D targeting strategies could be therapeutically beneficial for RA patients.

Similarly to the described above rheumatoid arthritis, the cell surface expression of Sema4D and serum sSema4D levels were significantly increased in MRL/lpr mice (an autoimmune murine model of lupus) when compared with those in MRL/n mice (Wang et al., 2001). The sSema4D levels directly correlated with the anti-ssDNA auto-Ab levels in MRL/lpr mice. As auto-Abs are involved in autoimmune disease pathogenesis, the sSema4D level could be a good diagnostic and prognostic marker for disease progression and the monitoring of medication effects.

Sema4D also plays an important role in many processes of neurodegeneration, and its inhibition may play a therapeutic role in different CNS diseases such as multiple sclerosis (EAE model of disease is discussed above) and Huntington disease. The YAC128 mice, as an experimental animal model, display the striatal, cortical, and corpus callosum atrophies and testicular degeneration which are the neuropathological signatures of Huntington disease (Southwell et al., 2015). The Anti-Sema4D Ab treatment of YAC128 mice significantly improved all these signatures in addition to behavioral symptoms such as anxiety-like behavior and cognitive deficits. This suggests that interfering with Sema4D-mediated pathway(s) could significantly improve the outcome of neurodegenerative diseases by hindering degeneration of nervous tissue.

Increased levels of both soluble and membrane-bound Sema4D were reported in serum and on T cells in patients with heart failure, where inflammation plays a significant role in pathogenesis and where Sema4D has been shown to participate in platelet activation as well as in thrombus formation in vivo (Lu et al., 2013). In addition to its main source of shedding - T cells, Sema4D has been shown to shed from activated platelet surface.

Consequently, Sema4D plays a critical regulatory role in many pathologic processes; this makes it a molecule of interest for potential immunotherapies of many immune-mediated diseases.

### Sema6D

#### Identification and structure

Molecular cloning, mapping, and functional analysis of Sema6D together with Sema6C have been carried out more recently compared to other semaphorins with costimulatory properties (Qu et al., 2002). The authors of this study isolated five full-length cDNA sequences, which represented a single novel human gene. Amino acid sequence alignment analysis of human semaphorin (HSA)SEMA6C, rat Sema6C, and mouse Sema6C showed the existence of the class VI semaphorin characteristic of extracellular domain and PSI domain, which differ from all known members of semaphorin family. Predicted structure (HSA)SEMA6D isoforms were compared with related semaphorin proteins. Sema6D consists of a signal peptide, a PSI domain, a transmembrane segment, an Ig domain, and a sema domain. For the (HSA)SEMA6D gene, the SEMA6D isoform is composed of only the first 13 exons. Each of the four long isoforms contains four to six additional exons at the third region and, interestingly, uses a cryptic acceptor site in exon 13 (described as exon 13a). In detail, exons 17 and 18 are deleted in SEMA6D.1; exons 16a, 17, and 18 are deleted in SEMA6D.2; exons 16a and 18 are deleted in SEMA6D.3; and exons 16a is deleted in SEMA6D.4. Four distinct long Sema6D isoforms were 5914, 5875, 5932, and 6100 bp in size and encoded 1011, 998, 1017, and 1073 aa, respectively. Sequence analysis has shown that the translated polypeptides are composed of a 1–21 aa signal peptide followed by a 59–477 aa sema domain, a 508–563 aa PSI domain, a transmembrane segment, and a long cytoplasmic region.

#### Expression

Although Sema6D mRNA was detected in all human tissues and organs (http://www.genecards.org/cgi-bin/carddisp.pl?gene=SEMA6D), the relatively high protein levels were identified in the parathyroid glands, heart muscle, gallbladder, pancreas, stomach, intestinal tract, testis, prostate, uterus, and fallopian tubes (http://www.proteinatlas.org) (Table 1). Sixteen splice variants have been reported, and the expression of distinct isoforms is thought to be regulated in a tissue- and development-dependent manner. Sema6D exists in both, membrane-bound and soluble forms (Toyofuku et al., 2004a).

#### Function

**Fig. 5:**
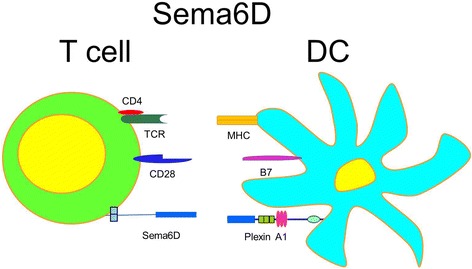
Sema6D acts as indirect T cell costimulatory molecule. T cell expressed Sema6D activates DC through Plexin A1 receptor. Polyclonally- or Ag-stimulated T cells upregulate Sema6D expression. Sema6D stimulates T cell viability, proliferation and cytokine production on late stages of immune response

Initially, O’Connor and associates (O'Connor et al., 2008) examined the regulation of T cell activation by Sema6D in vitro and in vivo. Upon T cell activation, after an initial decrease in Sema6D mRNA expression, they observed its stable upregulation and, later, a protein expression on the surface of T cells. This upregulation was relevant to both anti-CD3/CD28-stimulated and Ag-stimulated T cells (Fig. 5). Using Sema6D-Ig, the authors identified Plexin A1 on DC as a Sema6D receptor (Fig. 5). Interestingly, when anti-Sema6D blocking Ab was added to the cell cultures, it affected T cell proliferation on late stages (4–6 days of culture) whereas in the early stages (2–4 days), T cell viability and proliferation as well as cytokine (IL-2) production were not different from those without Ab in the culture (Fig. 5). Specific targeting of Sema6D decreased T cell activation in vivo in the OTII cell adoptive transfer model. In this model, OTII T cells were obtained from a TCR-transgenic strain that contains rearranged TCR-Vα and -Vβ genes in the germline DNA encoding a TCR specific for chicken ovalbumin (OVA) peptide 323–339 bound to I-A molecules in a context of H-2b haplotype (CD45.2). These CD45.2 cells were adoptively transferred to congenic B6-Ly5.2/Cr (CD45.1) recipients. The splenocyte proliferation assessed as an expansion of donor OTII T cells in the recipient mice and expressed as the percentage of TCR+ CD4+ CD45.2+ cells in the isolated splenocyte population. The donor cell numbers were significantly lower when the recipient (CD45.1) mice received Sema6D-Ig at the time of cell plus OVA protein injections. Interestingly, Sema6D-Ig treatment did not affect an early T cell activation (day 4) but significantly reduced CD45.2+ T cell expansion at day 7. It is still unclear, however, if Plexin A1 is the only functional receptor for Sema6D on DC. Moreover, Plexin A1 has other ligands (class III semaphorins), at least one of them being expressed by immune cells (Sema3A), which makes the final conclusion about Sema6D-PlexA1 interaction rather difficult. In addition, Plexin-A1 has been reported to form a receptor complex with vascular endothelial growth factor receptor type 2 (VEGFR-2) in the conotruncal segment or with off-track binding in the ventricle segment during cardiac morphogenesis (Toyofuku et al., 2004a). However, the presence of such a functional Plexin A1/VEGFR-2 complex on DC has not been checked yet. Takegahara and associates (Takegahara et al., 2006) have shown that an addition of a soluble Sema6D to the in vitro DC cultures induced DC activation as indicated by the IL-12 production and upregulation of cell surface MHCII expression. Those DC activation markers were significantly lower in PlexinA1^−/−^ DC cultures with rSema6D. In a search for a functional Sema6D receptor, the performed immunoprecipitation studies revealed that Trem-2 acts as a bridge between PlexinA1 and DAP12 and such functional complex is being formed in DC after Sema6D ligation. However, for Sema6D-PlexinA1 induced Rac activation DAP12 was dispensable. Further studies will clarify the mechanisms of immune response regulation by Sema6D. Moreover, as a transmembrane molecule Sema6D could also trigger a reverse signaling in immune cells as this capacity has been shown previously for myocardial cells (Toyofuku et al., 2004b). Because this semaphorin molecule has been discovered relatively recently, its organ- and tissue-specific functions so far have not been well characterized.

#### Disease relevance

A recent study in breast cancer patients has demonstrated that the expression of the major tumor metastatic promoter (MMP-) 9 was dramatically reduced among SEMA6D-high samples, while the levels of other tumour metastatic promoters such as TGF-β-related factors, ZEB2, ZEB1, and GNG11 directly correlated with levels of SEMA6D (Chen et al., 2015). The authors also admit a high correlation between SEMA6D and VEGF-family gene levels in breast cancer patients, suggesting a role of the VEGF family in mediating SEMA6D signaling. Further database analysis showed that increased expressions of SEMA6D, CLEC9A, COL4A6, and C10orf107 were associated with improved survival rates among breast cancer patients.

### Sema7A

#### Identification and structure

Sema7A, also known as the John-Milton-Hagen (JMH) blood group antigen, Sema-K, CD108, is an 80-kD glycoprotein expressed on activated lymphocytes and erythrocytes. It has been cloned and characterized by several research groups (Mudad et al., 1995; Lange et al., 1998; Yamada et al., 1999). Originally, it was identified as a vertebrate homolog of the viral A39R and AHV as the respective genes for viral semaphorins were cloned, sequenced, and termed H-Sema-L and M-Sema-L (SEMAL and Semal, respectively) (Lange et al., 1998). The multiply spliced mRNA for Sema7A, with the size of 3.2 kb, was found to be expressed in human reproductive and immune tissues. The human Sema7A gene maps to chromosome 15q22.3–q23, whereas its mouse gene maps to the homologous locus 9A3.3-B in the mouse genome. The expression patterns and the presence of related genes in large DNA viruses let the researchers to suggest that this new semaphorin has a relevant function in the immune system.

The cDNA clone containing the entire coding sequence of the Sema7A gene and its molecular characteristics were first reported by Yamada and associates (Yamada et al., 1999). As a first step, human Sema7A cDNA clones were identified through the screening of a plasmid library generated from a leukemic T cell line. The 1998-base pairs of the cloned DNA’s open reading frame encoded a 666 aa protein. This protein contained a 46 aa signal peptide and a 19 aa GPI-anchor (glycophosphatidylinositol linkage) motif. The membrane-anchoring form of Sema7A was the 602 aa. The estimated molecular mass of the unglycosylated form was 68 kDa. The authors located an “RGD (Arg-Gly-Asp) cell attachment sequence and the five potential N-linked glycosylation sites on the membrane-anchoring form”. Immunoprecipitation and flow cytometry analyses of cell transfectants confirmed the expression of a native Sema7A form. The Sema7A gene was pinpointed to the middle of a long arm of chromosome 15 (15q23–24) by radiation hybrid mapping. The detailed molecular characteristics of Sema7A provided in the article by Yamada and associates (Yamada et al., 1999) was critical for understanding the Sema7A biological function and for further research involving this neuroimmune semaphorin function. The murine Sema7A cDNA is highly homologous to human Sema7A (88.0% similarity at the nucleotide level or 89.3% similarity at the amino acid level of protein) (Mine et al., 2000). Both human and mouse SEMA7A contain a seven-bladed β-propeller semaphorin N- terminus domain, a plexin, semaphorin, and integrin domain (PSI), an immunoglobulin-like domain, and the characteristic for this particular semaphorin molecule GPI anchor in their C-terminus (Liu et al., 2010). SEMA7A can dimerize through the Sema and immunoglobulin domains (Liu et al., 2010). Initially, it had been reported that Sema7A binds Plexin C1 receptor with high affinity (Tamagnone et al., 1999), however, such binding had not been functionally proven to exist in monocytes (Holmes et al., 2002). Later, such unusual side-to-side binding between the two molecules has been defined in details (Liu et al., 2010). Sema7A functionally ligates two receptors of the integrin family such as a very late antigen 1 (VLA-1) dimer, α1β1 integrin (Pasterkamp et al., 2007), and αvβ5 integrin (Kang et al., 2012). Adding to the complexity of the semaphorin system, α1β1 integrin is a major collagen-binding protein that allows cells to bind to collagenous substrates and grow on them (Pozzi et al., 1998), while integrin αvβ5 also serves as a receptor for vitronectin, a 75-kd plasma glycoprotein involved in cell adhesion and spreading and in coagulation pathways (Preissner, 1991). Thus, the Sema7A effect could be distinct in different inflammatory diseases due to the expression levels of different integrin ligands; however, it is still unknown if there is a receptor-binding competition for different integrin ligands.

#### Expression

The Sema7A mRNA was expressed in activated PBMCs as well as in the spleen, thymus, testis, placenta, and brain (Table 1) (Yamada et al., 1999). Later, Holmes and associates (Holmes et al., 2002) reported the highest expression of Sema7A mRNA in macrophages, neutrophils, C13 cells (immortalized immature microglial cells), as well as lung and brain tissues. Sema7A protein was found to be expressed in bone marrow, the immune system, lungs, the pancreas, the brain, the gastrointestinal tract, and the female reproductive system (the Human Protein Atlas (http://www.proteinatlas.org)). Lower levels of protein expression were found in skin, male reproductive tissues, muscle, and endocrine tissues.

In the neuroendocrine system, the expression pattern of two Sema7A receptors, Plexin C1 and α1β1 integrin, is spatiotemporally regulated: at early stages of development, migrating gonadotropin releasing hormone (GnRH) neurons only express α1β1 integrin, whereas during subsequent stages, they start to express Plexin C1 and stop migrating (Parkash et al., 2015).

#### Function

**Fig. 6:**
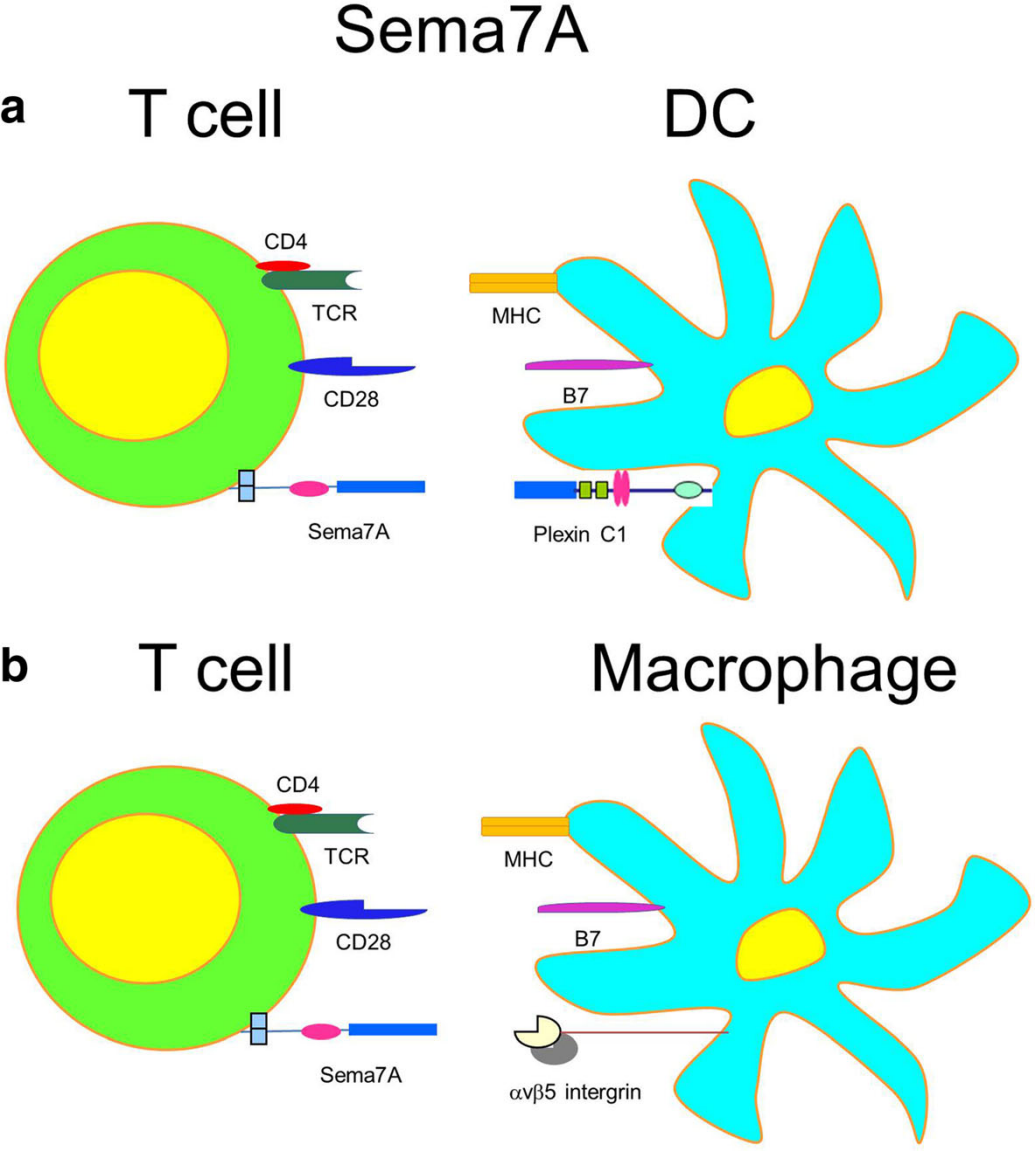
T cell inducibly expressed Sema7A has distinct receptor-dependent effects on DC and macrophages. **a** Sema7A in T cell-DC interaction. Indirect T cell stimulation by T cell expressed Sema7A ligation of Plexin C1 on DC. **b** Sema7A in T cell-macrophage interaction. Inflammatory macrophages get activated and stimulated for pro-inflammatory cytokine secretion by Sema7A ligation of α1β1 integrin

Sema7A interaction with α1β1 integrin is critical for axonal growth (Parkash et al., 2015; Pasterkamp et al., 2003), whereas epithelial Sema7A binding to macrophage αvβ5 integrin is crucial for anti-inflammatory IL-10 secretion in a colitis model (Kang et al., 2012). Similarly to Sema6D, Sema7A is expressed only on activated T cells and stimulates macrophages through α1β1 integrin receptor for proinflammatory cytokine production (Suzuki et al., 2007) (Fig. 6). Therefore, Sema7A is an inducible indirect costimulatory molecule for T cell activation through APC. The molecular basis for interaction between Sema7A and integrins includes the Arg-Gly-Asp (RGD) attachment sequence as a prototypical binding motif for β-integrins which is also found in extracellular matrix proteins (Pasterkamp et al., 2003). This RGD-intergin connection is also a major recognition system for cell adhesion. In contrast to a Sema7A-mediated dose-dependent growth-promoting effect on olphactory neurons, Sema3A did not display such effect despite having an overall similar structure but missing RGD motif (Pasterkamp et al., 2003). Therefore, integrin-mediated signaling is critical in Sema7A-mediated axon guidance. Holmes and associates (Holmes et al., 2002) have shown that recombinant Sema7A had no effect on inducing T cell proliferation or on Th1/Th2 cytokine production independently of the presence of specific T cell activators. Sema7A also had no effect on proinflammatory cytokine production by B cells (Holmes et al., 2002). However, the low doses of Sema7A in monocyte cell cultures have been shown to stimulate the production of proinflammatory cytokines (IL-1β, TNF-α, IL-6, IL-8) in a dose-dependent manner (Fig. 6). Moreover, the presence of Sema7A within the lipid rafts at the immunological synapse between T cells and macrophages also proves its importance as a costimulatory molecule for APC.

The seminal paper by Czopik and associates (Czopik et al., 2006) extensively examined a costimulatory role of Sema7A and demonstrated that it acts as an inhibitor of T cell activation and essential constrainer of T cell-mediated autoimmunity. Sema7A^−/−^ T cells demonstrated an enhanced proliferation upon Ag re-stimulation in vitro. This increased proliferation was dependent on a lack of Sema7A expression on T cells but not on APC. Moreover, Sema7A deficiency in DC did not affect either in vitro TLR ligand-induced DC maturation or their ability to activate WT T cells. Based on the extensive in vitro and in vivo T cell analysis, the authors proposed a model of Sema7A function in T cell signaling where Sema7A interacts with the components of TCR complex and with a putative receptor on APCs what stabilizes TCR/CD3 complex and promotes inhibitory signals that limit T cell proliferation.

Sema7A has been shown to promote the cell dendricity (Pasterkamp et al., 2003; Suzuki et al., 2007; van Rijn et al., 2016) and, thus, to drive monocytes into the CD11b+/CD14−/CD1α−/CD83+/CD40+/CD86+ DC-like phenotype (Holmes et al., 2002). In addition, it has been shown recently that Sema7A expression on DC was not required for DC maturation but was necessary for DC migration in response to CCL21 (van Rijn et al., 2016). This suggests its critical role in DC guiding to lymphatic vessels and into lymph nodes. Again, adding to the complexity of individual semaphorin interaction with multiple receptors displayed on multiple cell types, Sema7A has been shown to bind Plexin C1 (Liu et al., 2010) which is expressed on murine activated T cells and DC (Walzer et al., 2005; Walzer et al., 2005) as well as on human monocytes (Chabbert-de Ponnat et al., 2005). Although Plexin C1 deficiency on DC did not affect the in vitro T cell response to Ag, the in vivo T cell response was reduced (Walzer et al., 2005). This particular study did not examine the effect of Sema7A-PlexC1 interaction in T cell response. Therefore, the effect of DC-expressing Sema7A on DC migration (van Rijn et al., 2016) can be mediated by two known Sema7A receptors, both expressed by DC, α1β1 integrin and Plexin C1, which still needs to be determined.

Sema7A has been shown to play a critical role in GnRH neuron-dependent reproductive competency (Parkash et al., 2015). GnRH-releasing neurons project their axons into the specific part of hypothalamus – the median eminence (ME) - the interface between the neural and peripheral endocrine systems. In this area, the neurons secrete GnRH to the blood vessels. GnRH is being then delivered to the pituitary glands and induces the production of luteinizing hormone and follicle-stimulating hormone. The special glial cells, tanycytes, have been shown to insulate the pericapillary space of pituitary portal vessels from the GnRH nerve terminals. Tanycytes express Sema7A and its expression is dynamically regulated during the oestrous cycle by fluctuating gonadal hormone levels. Therefore, Sema7A expression levels highly depend on the hormonal state. Cyclically released from tanycytes Sema7A induces GnRH neurons to retract their terminals away from the pericapillary space through PlexinC1 binding and signaling. In addition to that, Sema7A promotes tanycytic end-feet expansion via α1β1 integrin what makes the pericapillary space inaccessible to GnRH nerve terminals. Altogether, these studies shed light on the molecular mechanisms responsible for the progression of the oestrous cycle.

#### Disease relevance

The significance of the Sema7A signaling pathway for T cell activation through activation of APC has been demonstrated in the in vivo studies in the contact hypersensitivity (CHS) and EAE animal models (Suzuki et al., 2007). Typical for CHS reaction, ear swelling and mononuclear cell infiltration were abrogated in Sema7A^−/−^ mice as compared to WT mice, which were sensitized and challenged with 2,4-dinitrofluorobenzene (DNFB). Moreover, the MOG peptide-immunized Sema7A^−/−^ mice were highly resistant to the EAE induction as compared to WT counterparts. Cell transfer studies were performed to demonstrate that a Sema7A molecule on T cells was required for the effector phase of immune response. This phase demonstrated that DNFB-primed Sema7A^−/−^ T cells were unable to transfer CHS even to WT recipients.

Sema7A deficiency in mice did not affect cerebellar myelination but prevented LPS-induced in vitro neuro-degeneration and led to either a resistance (a low immunization dose of MOG_35–55_) or a milder form of the disease (a high immunization dose of MOG_35–55_), compared to similarly treated WT mice (Gutierrez-Franco et al., 2017). High dose Ag immunization led to a lower demyelination and infiltration of inflammatory cells in Sema7A-deficient brains as compared to that in WT littermates, which correlated with the milder clinical outcome. In contrast, the study by Czopik and associates (Czopik et al., 2006) have shown a dramatically increased MOG_35–55_ peptide- induced EAE disease severity index, morbidity, and delayed recovery in Sema7A^−/−^ mice as compared to WT littermates. These clinical alterations were associated with an increased T cell infiltration to CNS and an enhanced CD4+ T cell re-call responses to Ag in vitro observed in Sema7A^−/−^ mice which were efficiently inhibited when soluble recombinant Sema7A was added to the cell cultures. Apparently, Sema7A has an autonomous inhibitory effect on T cell activation as there was a robust Sema7A^−/−^ T cell expansion (50% of total spleen cells as compared to 2–3% with transferred CFSE-labeled WT T cells) in lymphopenic RAG2^−/−^ host. These Sema7A-deficient cells also showed increased expression CD25 activation marker as compared to Sema7A^+/+^ T cells. Ultimately, the authors of both studies suggested that Sema7A might be a potential therapeutic target to treat multiple sclerosis (MS) and other autoimmune conditions.

The role of Sema7A in lung injury and pulmonary inflammation was examined by Roth and associates, using molecule-deficient mice and lung LPS exposure model (Roth et al., 2016). The authors reported a positive loop between Sema7A and inflammation. They described that lung inflammation induces an upregulation of a local Sema7A expression. Moreover, they found that Sema7A expression itself regulates the pro-inflammatory response in the lung tissue. Sema7A is induced in vitro in endothelial cells by pro-inflammatory cytokines such as IL-6 and TNF-α. The exposure of endothelial and epithelial cells with recombinant Sema7A induces these same pro-inflammatory cytokine secretion through a direct NF-kB activation. Besides, the authors have demonstrated that Sema7A, which is released from endothelial and epithelial cells during lung injury, acts as a chemo-attractant for neutrophils. In general, this study shows the importance of Sema7A for the control of an acute inflammatory response and the treatment of acute lung injury. The interference with the described pathway holds the potential to modulate acute inflammation in the future.

Sema7A expression in CNS on oligodendrocyte progenitor cells as well as in mature oligodendrocytes and immune cells is upregulated during EAE progression (Gutiérrez-Franco et al., 2016). This, together with other discussed above studies (Suzuki et al., 2007; Czopik et al., 2006), supports Sema7A involvement in disease pathogenesis and its potential as a therapeutic target in MS. Indeed, the analysis of Sema7A, α1-integrin, and β1-integrin expression in demyelinated MS lesions has shown a strong Sema7A staining in reactive astrocytes while microglia/macrophages only expressed β1-integrin (Costa et al., 2015). The levels of Sema7A expression directly correlated with the levels of inflammation in the MS lesions, suggesting its direct contribution to the impairment of tissue regeneration and regulation of immune response. The levels of soluble Sema7A were significantly lower in the cerebrospinal fluid of patients with clinically isolated syndrome (CIS) who converted to clinically definite MS (CDMS) as compared to those who did not convert (Canto et al., 2014). Specific clinical and radiological criteria used to classify CIS patients into MS converters and nonconverters were similar to ones defined in the previously performed proteomic study. In this research study, the authors demonstrated that Sema7A can serve as a predictive biomarker associated with a conversion to CDMS in CIS patients. The authors concluded that decreased CSF levels of Sema7A observed in MS converters may be related to its role as a negative regulator of T-cell activation. Sema7A^−/−^ mice showed a more severe course of EAE disease with exacerbated nervous tissue pathology, and Sema7A^−/−^ T cells showed increased proliferative responses to a disease-inducing agent, MOG Ag. As for the allergic disease, it has been shown that Sema7A mRNA level increased 163-fold in BAL cells after a segmental antigen challenge compared to BAL cells obtained from the same subjects before allergen challenge (Esnault et al., 2014).

In order to understand the role of Sema7A in breast cancer progression, its expression levels were determined for normal breast tissues and compared with invasive ductal carcinomas (IDC) using the TCGA dataset and Oncomine software (Black et al., 2016). Sema7A expression was significantly increased in IDC compared to normal tissue. Moreover, there was a direct correlation between Sema7A levels and early cancer recurrence, metastasis, and patients’ patient’s deaths in multiple publicly available datasets for breast cancer patients. Importantly, the analysis of these datasets indicated that the increased Sema7A gene expression was directly associated with a poor prognosis. The authors also examined the effects of Sema7A silencing using shSema7A as a potential anti-tumor therapeutic measure. The presented data clearly demonstrated that reduced Sema7A levels were associated with decreased human breast tumor cell growth, motility, invasion, and adhesion. In addition, the authors identified a novel stimulatory role for Sema7A in lymphangiogenesis and defined the mechanism of Sema7A action in breast cancer as being regulated by COX-2 and activating β1-integrin receptor expression, which mediated tumor cell invasiveness. The loss of Sema7A expression by tumor cells reduced their potential for invasion and their ability to activate β1-integrin receptor-related signaling pathways. Cumulatively, the presented data suggest that Sema7A-related therapies could be beneficial for breast cancer patients. Further developments in this area of research may lead to discoveries of novel anti-cancer therapeutics.

## Conclusion

This review focuses on the actions of neuroimmune semaphorin as costimulatory molecules for either direct or indirect T cell activation and immune response modulation. It also provides brief discussions of current reports delineating neuroimmune semaphorins’ identification, structures, corresponding receptors, cell/tissue/organ expression, and function in different diseases. Each neuroimmune semaphorin has multiple receptors expressed in various tissues and performs a multitude of functions. However, the data discussed here demonstrate that the manipulation of neuroimmune semaphorins and/or their receptor expressions and/or corresponding signaling pathways could become attractive therapeutic approaches for many diseases, especially as a combination therapy with other agents. Further studies are warranted for future development as the signaling pathways that regulate the expression of the aforementioned semaphorins and their corresponding receptors, as well as the signaling pathways involved in semaphorin-receptor actions, are either under investigation or remain unknown.
